# Improvements in PPP by Integrating GNSS with LEO Satellites: A Geometric Simulation

**DOI:** 10.3390/s25144427

**Published:** 2025-07-16

**Authors:** Marianna Alghisi, Nikolina Zallemi, Ludovico Biagi

**Affiliations:** DICA Politecnico di Milano, Piazza Leonardo Da Vinci 32, 20133 Milano, Italy; marianna.alghisi@polimi.it (M.A.); nikolina.zallemi@polimi.it (N.Z.)

**Keywords:** GNSS, LEO-PNT, PPP, hybrid positioning, PDOP

## Abstract

The precise point positioning (PPP) method in GNSS is based on the processing of undifferenced phase observations. For long static sessions, this method provides results characterized by accuracies better than one centimeter, and has become a standard practice in the processing of geodetic permanent stations data. However, a drawback of the PPP method is its slow convergence, which results from the necessity of jointly estimating the coordinates and the initial phase ambiguities. This poses a challenge for very short sessions or kinematic applications. The introduction of new satellites in Low Earth Orbits (LEO) that provide phase observations for positioning, such as those currently provided by GNSS constellations, has the potential to radically improve this scenario. In this work, a preliminary case study is discussed. For a given day, two configurations are analyzed: the first considers only the GNSS satellites currently in operation, while the second includes a simulated constellation of LEO satellites. For both configurations, the geometric quality of a PPP solution is evaluated over different session lengths throughout the day. The adopted quality index is the trace of the cofactor matrix of the estimated coordinates, commonly referred to as the position dilution of precision (PDOP). The simulated LEO constellation demonstrates the capability to enhance positioning performance, particularly under conditions of good sky visibility, where the time needed to obtain a reliable solution decreases significantly. Furthermore, even in scenarios with limited satellite visibility, the inclusion of LEO satellites helps to reduce PDOP values and overall convergence time.

## 1. Introduction

The term global navigation satellites systems (GNSSs) refers to all the constellations of artificial satellites operated by national or international spatial agencies for positioning, navigation and timing (PNT) applications. Currently, four GNSS constellations are operational at a global scale: the United States’ GPS, the European Galileo, the Russian Glonass and the Chinese BeiDou [1,2,3]. With the exception of BeiDou’s geostationary (GEO) and inclined geosynchronous (IGSO) satellites [4], the core GNSS constellations operate in Medium Earth Orbit (MEO). These orbits are nearly circular, with orbital radius comprising between 25,510 km (Glonass) and 29,994 km (Galileo) and an approximate orbital velocity of 4 km/s. All GNSS satellites provide two types of observations for PNT: code observations, also referred to as pseudoranges, and carrier-phase observations. All of them provide observations on two or three frequencies. Multiple techniques are available for processing GNSS observations for PNT services, with each one targeting different accuracy requirements and application needs. These techniques range from single-point positioning (SPP), which provides meter-level accuracy suitable for general navigation, to more advanced methods such as precise point positioning (PPP) and real-time kinematic (RTK), which can achieve centimeter- or even millimeter-level precision. As defined in [5], SPP represents the most basic GNSS processing strategy, relying only on code observations to estimate both the position and clock offset of the receiver [1]. This technique is typically implemented for real-time applications, where satellite coordinates and clocks are given by navigational messages. SPP can be implemented in either single-frequency or in multi-frequency modes. In the first case, ionospheric effects must be modeled, whereas in the second case, an ionospheric-free combination of signals can be built and processed. Under favorable conditions, such as open-sky environments, the main error sources in SPP are the residual errors in the navigation message, in the modeling of atmospheric effects and electronic noise, typically at the decimeter level for high-quality receivers. Consequently, SPP solutions generally achieve meter-level positioning accuracy [5]. However, in challenging scenarios, such as urban canyons, the presence of buildings and reflective surfaces causes non-line-of-sight (NLoS) and multipath effects [6,7]. Therefore, in such conditions, the accuracy of an SPP solution degrades, and, in extreme cases, the positioning solution may become unavailable due to an insufficient number of visible satellites, rendering the system unsolvable—i.e., fewer satellites than the number of unknowns. To obtain precisions of a few centimeters or better, the processing of phase observations is needed. In a nutshell, two philosophies are possible: the differential and undifferenced approaches [8]. In the first one, the so-called double differences between pairs of receivers are constructed and processed. This approach is well established, both for the processing of permanent monitoring networks and for fast static and kinematic applications. A particular application is that of real-time kinematic (RTK): RTK is a high-precision differential GNSS technique that achieves centimeter-level accuracy by applying real-time corrections from reference stations [9]. RTK is effective within a range of 10–20 km from a reference station and is widely used in surveying applications. However, it presents limitations related to the availability of reference stations, the quality of the communication link, and the need for continuous signal tracking. The alternative approach is the undifferentiated processing of phase observations, which is called precise point positioning. PPP improves positioning accuracy by using precise satellite orbit and clock corrections obtained from external services, rather than relying solely on the broadcast navigation message [10]; by a joint processing of code- and carrier-phase measurements, it allows for centimeter- to decimeter-level accuracy under favorable conditions [5]. At present, the PPP technique is well-established in open-sky scenarios, with a good satellite visibility, and it is widely adopted for static post-processing. Recent studies have demonstrated the feasibility of kinematic and real-time PPP implementations [11,12]. However, PPP implies the joint estimation of coordinates and ambiguities in carrier-phase measurements [13], and requires tens of minutes to achieve full accuracy: this relatively long convergence time poses serious limits to real-time applications. Moreover, significant challenges remain in obstructed environments, like urban canyons, where the limited satellite visibility increases significantly the convergence time. In this study, we focus on PPP in a batch processing context, simulating a least squares (LS) solution applied to a set of GNSS observations and simulated Low Earth Orbit (LEO) observations. This approach, commonly adopted in geodetic data processing, can also serve as an effective method for navigation system initialization. The batch processing method provides a stable and robust solution by leveraging an extended set of observations to mitigate measurement noise and enhance positioning accuracy. The aim of this paper is to investigate PPP processing in open-sky and obstructed scenarios. In particular, we investigate the geometric improvement introduced by a simulated LEO-PNT new constellation. Our focus is the time needed to obtain a fair geometry, that is the convergence time. As the statistical index, we will consider the trace of the cofactor matrix of the coordinates estimates, obtained by the simulations. Note that this index is equal to the position dilution of precision (PDOP) index, normally used to characterize the geometry quality in the single-epoch SPP. For this reason, we will use the term PDOP. This index will be used to quantify the time required to achieve a satellite geometry that meets the condition of PDOP ≤1. In this work, we simulate the orbits of a LEO-PNT constellation, designing it in line with the existing literature on LEO-PNT systems. Unlike previous studies, which use the orbital parameters of existing satellite communication (satcom) constellations, this work proposes and evaluates a constellation specifically conceived for navigation purposes. At present, since operational LEO constellations intended for navigation are not yet available, the simulation of geometries and the following computation of the PDOP is the only practical way to assess the expected improvements by integrating new LEO-PNT satellites to an existing GNSS constellation. PDOP alone does not directly quantify the positioning final accuracy, which is clearly affected by several other phenomena, in particular multipath in urban scenarios; however, it provides valuable insights into the potential reduction in convergence time by enhancing satellite geometry.

### GNSS Positioning Model

In PPP, both carrier-phase observations and pseudo-range (code) observations are processed together [1]. Following the conventional notation in [10], the code observable from satellite *s*, of constellation *C*, on frequency *j* to receiver *r* is expressed as follows:

(1) $$\begin{array}{cc} P_{r,j}^{s}\left(t\right)= & \rho_{r}^{s}\left(t,t-\tau_{r}^{s}\right)+T_{r}^{s}\left(t\right)+c\left[dt_{r}\left(t\right)+d_{r,j}^{C}\left(t\right)\right]-c\cdot dt^{s}\left(t-\tau_{r}^{s}\right)\\ & +\mu_{j}I_{r}^{s}\left(t\right)+\epsilon_{r,j}^{s}\left(t\right) \end{array}$$

The carrier phase observable from satellite *s* to receiver *r* is expressed as:

(2) $$\begin{array}{cc} {Ф}_{r,j}^{s}\left(t\right)= & \rho_{r}^{s}\left(t,t-\tau_{r}^{s}\right)+T_{r}^{s}\left(t\right)+c\left[dt_{r}\left(t\right)+\delta_{r,j}^{C}\left(t\right)\right]-c\cdot dt^{s}\left(t-\tau_{r}^{s}\right)\\ & -\mu_{j}I_{r}^{s}\left(t\right)+\lambda_{j}^{s}\eta_{r,j}^{s}+\epsilon_{r,j}^{s}\left(t\right) \end{array}$$

where: $\rho_{r}^{s}$ is the pseudo-distance between the receiver and the satellite; $T_{r}^{s}$ is the tropospheric delay; $\mu_{j}I_{r}^{s}$ is the ionospheric delay; $dt_{r}$ is the receiver clock offset; $d_{r,j}^{C}\left(t\right)$ is the receiver code intersystem bias (ISB) for constellation *C*, $\delta_{r,j}^{C}\left(t\right)$ is the receiver phase ISB for constellation *C*; $dt^{s}$ is the satellite clock offset with respect to the relative system time; $\lambda_{j}^{s}$ is the wavelength; $\eta_{r,j}^{s}$ is the carrier-phase ambiguity. $\epsilon_{r,j}^{s}$ is the observation error. Phase observations have an electronic noise in the order of mm; that is, about two orders of magnitude smaller than code observations. This leads to a potential accuracy consistent with high-precision applications. However, the inclusion of carrier-phase observations introduces an unknown initial phase ambiguity for each satellite, thus resulting in the need of a batch processing of more epochs [14]. Furthermore, the technique requires careful data screening for cycle slips [2]. To fully exploit the accuracy of phase observations, all the residual errors of real-time SPP must be removed. Moreover, the need to jointly estimate coordinates and ambiguities implies a slow convergence of the solution [13]. These two limitations do not represent a problem for post processing of static surveys, where long continuous sessions allow PPP solutions to converge. For this reason, PPP is a standard approach for processing data from continuously operating reference stations, using final orbits, satellite clocks and biases published by the International GNSS Service (IGS) [15]; see, for example, [16,17]. On the contrary, real-time or kinematic PPP applications remain a topic of ongoing research. Some emerging solutions, such as quasi-real-time processing via the Galileo High-Accuracy Service (HAS), are now in progress [18,19]. These developments, while valuable, are not the focus of this study. Clearly, the possibility to use the PPP approach for kinematic applications will become important in the future, considering the present availability of low-cost receivers that provide both code and phase multi-frequency observations [20]. This paper specifically focuses on the problem of the convergence of the PPP solution and the potential advantages offered by integrating a future LEO-PNT constellation with the existing GNSS constellations. Section 2 presents, in general, the LEO-PNT constellations; at the end delineates the scenario more in details. Section 3 discusses the least squares method used to simulate the processing. Section 4 presents the simulation setup. Section 5 presents the numerical results.

## 2. Low-Earth-Orbiting Satellites for PNT

As previously stated, PPP requires the joint estimation of receiver coordinates and phase ambiguities. In this context, the solution convergence time remains a major limitation. This is primarily due to the slow geometric variation of GNSS satellites relative to a ground-based receiver, as these satellites operate at altitudes of approximately 20,000 km and velocities of about 4 km/s [13]. In a harsh urban environment, the visibility and geometry of the satellites are compromised, affecting their overall performance or, in extreme cases, making convergence impossible. Over the past few decades, several LEO satellite constellations—typically orbiting at altitudes between 400 km and 2000 km—have been deployed for various purposes, such as telecommunication and Earth observation [21,22,23]. Due to their proximity to the Earth, LEO satellites require less energy to launch, lowering deployment costs. Several agencies, both private and public, are now exploiting the potential of designing and using LEO satellites for PNT purposes, equipping them with GNSS-like instruments and signals. In this context, LEO-PNT constellations can change this scenario by enhancing traditional GNSS-based positioning. Their contribution lies both in increasing the system redundancy and improving geometric diversity. LEO satellites orbit at velocities ranging from 7 km/s to 8 km/s, completing an orbit in approximately 90 min to 2 h. This high orbital speed results in rapid changes in satellite–receiver geometry, which can, in principle, reduce the convergence time of PPP solutions [24,25]. This rapid movement necessitates the use of constellations—networks of interconnected satellites that collectively provide consistent global coverage and robust real-time data transmission. These constellations, featuring multiple orbital planes and intersatellite links (ISLs), enable direct communication between satellites without the need for ground stations, thus improving signal resilience and reducing atmospheric interference [26]. This study aims to analyze the impact of integrating LEO-PNT constellations with traditional GNSS on PPP performance in fast static and kinematic sessions. A least squares estimation is used to evaluate the solution convergence, focusing on the conditioning and stability of the related normal matrices. The analysis begins by computing the PDOP values using only GNSS data, for different time intervals, in a batch PPP solution, using final ephemerides provided by IGS [15]. The simulated LEO constellation follows examples available in the literature ensuring consistency with established models [22,23,27]. Specifically, the constellation is computed using a Keplerian model with circular orbits. Global coverage is achieved through the use of polar orbits, and the satellite distribution consists of 263 satellites across 19 orbital planes [28]: 8 orbital planes inclined at $89^{\circ }$ to ensure global coverage, and 11 orbital planes inclined at $55^{\circ }$ to reinforce coverage across mid-latitudes, which correspond to the most densely populated regions of the globe. While several studies in the literature analyze LEO mega-constellations for SPP [25,29,30], these referenced works often consider commercial LEO constellations, such as Starlink, which are not specifically designed or optimized for PNT purposes. In contrast, this study employs a constellation model consistent with the existing European Space Agency (ESA) literature related to the LEO-PNT project, which considers system architectures tailored for PNT applications [31,32]. The analysis is conducted across three representative environments for PNT applications: open-sky, an urban scenario with typical residential buildings, and a dense urban canyon scenario.

## 3. Methods

In satellite-based positioning, the estimation of the position of a receiver from satellite observations leads to redundant systems. To solve such overdetermined systems, standard approaches include least squares (LS) estimation [5] or extended Kalman filtering (EKF) [33]. This work adopts a batch LS approach for simulation purposes. A test site is chosen as a case study, and two different satellites configurations are evaluated as follows:

1. Only GNSS available, by using the actual GPS/GALILEO/BeiDou satellites in orbit and in LoS,
2. GNSS + LEO, where LEO constellation (and visibility table) is simulated.

For both configurations, the quality of the PPP solution is investigated, checking the time needed for a static session to provide an acceptable solution for the position estimate. In particular, the simulations will be performed under the following working hypotheses:

1. Phase observations are processed,
2. Coordinates, clock offsets, and ISBs of the receiver are estimated, together with the initial ambiguities.

To evaluate the solution convergence, the PDOP index is used as a geometric indicator [34]. A brief summary of the mathematical model is given in the following section.

### 3.1. Least Squares Simulation Setup

In the LS model [35], given a vector $y_{0}$ of *m* observations affected by measurement errors, we can write

(3) $$y_{0}=y+\epsilon$$

where y is the vector of unknown observables and ϵ represents the measurement errors.

The observables are related to a vector x of *n* unknowns by a linear deterministic model, as follows:

(4) y=Ax+b

where A is called the design matrix, and b is a known term. The observations are assumed to follow a normal distribution as follows:

(5) $$y_{0}=N\left[y,C_{yy}\right],C_{yy}=\sigma_{0}^{2}Q_{yy}$$

where $C_{yy}$ is the covariance matrix of the observations, $\sigma_{0}^{2}$ is the a priori variance, $Q_{yy}$ is the so-called cofactor matrix of observations. Let us assume that A is of full rank. The LS solution is

(6) $$\hat{x}=N^{-1}A^{T}Q_{yy}^{-1}\left(y_{0}-b\right)$$

where N, called the normal matrix, is given by $N=A^{T}Q_{yy}^{-1}A$.

After the estimate of the unknowns, the observations, the residuals and a posteriori variance ${\hat{\sigma }}^{2}$ can be estimated. The final covariance matrix of the unknown parameters is given by

(7) $$C_{xx}={\hat{\sigma }}^{2}N^{-1}={\hat{\sigma }}^{2}Q_{xx}$$

where we name $Q_{xx}$ the cofactor matrix of the estimated parameters, and ${\hat{\sigma }}^{2}$ is the a posteriori variance factor, computed after estimation as:

(8) $${\hat{\sigma }}^{2}=\frac{{\left(y_{0}-A\hat{x}\right)}^{T}Q_{yy}^{-1}\left(y_{0}-A\hat{x}\right)}{m-n}$$

${\hat{\sigma }}^{2}$ depends on the quality of the input observations [36] and its discussion is outside the scope of this paper. On the contrary, $Q_{xx}$ depends only on A and $Q_{yy}$; therefore, the potential accuracy of a given scenario can be simulated and assessed just by building these two matrices. Different scenarios $S_{i}$ can also be simulated and compared by checking the respective $Q_{xx}$.

If A is rank-deficient (or almost rank-deficient), then N is singular (or ill-conditioned) and the system of Equation (6) cannot be trivially solved. In these cases, which are quite common in geodetic and geomatics problems, specific solutions can be adopted to regularize and solve them; see, for example, [37,38,39]. In the case of PPP, the convergence time is exactly the time needed to have a well-conditioned N. A possible measure is given by the so-called PDOP [34], which is discussed in the following section.

### 3.2. Application to Our Problem

In the following section, we simulate to process the ionospheric-phasefree combination; the tropospheric delay is assumed to be known by an a priori model, and the satellites clocks and biases are assumed to be known by precise ephemerides. The phase observation equation is linearized with respect to the unknown receiver coordinates; the receiver clock offset is grouped with the constellation ISBs. Recalling Equation (2), the reduced observation equation of ionospheric free is given by the

(9) $$\delta \phi_{r}^{s}\left(t\right)=\phi_{r}^{s}\left(t\right)-{\tilde{\rho }}_{r}^{s}-T_{R}^{S}+c\cdot dt_{s}\left(t\right)=e_{r}^{s}\delta x_{r}+c\left[dt_{r}^{C}\left(t\right)\right]+\lambda_{j}^{s}\eta_{r,j}^{s}$$

where $\delta \phi_{r}^{s}\left(t\right)$ is the reduced phase observation between the receiver *r* and the satellite *s* at time *t*, ${\tilde{\rho }}_{r}^{s}$ is computed by the approximate coordinates of *r* (${\tilde{x}}_{r}$) and the ephemerides coordinates of *s* ($x^{s}$), $T_{R}^{S}$ is assumed to be known from an a priori model, $dt^{s}\left(t\right)$ is assumed to be known from the ephemerides. $e_{r}^{s}$ is the unitary vector from receiver *r* to satellite *s*, defined as $e_{r}^{s}=\frac{{\tilde{x}}_{r}-x^{s}}{{\tilde{\rho }}_{r}^{s}}$.

Finally, $\delta x_{r}$ is the correction to the receiver’s approximated position, $dt_{r}^{C}\left(t\right)=dt_{r}\left(t\right)+\delta_{r}^{C}\left(t\right)$ is the combined receiver clock offset and constellation-dependent ISBs. Therefore, $\delta x_{r}$, $dt_{r}^{C}\left(t\right)$ and η are the unknown parameters in the LS system solution.

Note that the observation equations of phases are assumed to be equal for both GNSS and LEO. In the PPP LS solution, the design matrix A has the following structure:

(10) $$A=\left[\begin{array}{ccc} A_{p} & A_{c} & A_{a} \end{array}\right]$$

where $A_{p}$, $A_{c}$, $A_{a}$ are the submatrices of A that link the observations, respectively, to the coordinates of the receiver, to the clock offsets and to the ambiguities. In particular, all of them have as many rows as the number of observations; sub-matrix $A_{p}$ has three columns, and each row contains the relevant components of the vector $e_{r}^{s}$; sub-matrix $A_{c}$ has as many columns as there are observation epochs: each column contains ones for the observations of the relevant epoch, and zero elsewhere; sub-matrix $A_{a}$ has as many columns as there are the observed satellites: it contains ones for the observations of the relevant satellite, and zero elsewhere. By simulating a certain configuration of available GNSS or GNSS and LEO satellites for a certain measurement session, the computation of A is quite straightforward. For more technical details about the implementation of formulas and the building of the blocks of the design matrix, the reader is invited to refer to [40]. To compute $Q_{xx}$, we need to decide the structure of $Q_{yy}$: in the following simulations we simply put $Q_{yy}=I$. The resulting cofactor matrix $Q_{xx}$ of the unknowns can be computed by Equation (7). Note that, at the end, $Q_{xx}$ depend only on the geometry of the given problem; this allows a simulation of the potential accuracies even without available observations. Moreover, by maintaining the partition in position, clocks and ambiguity blocks (p,c,a subscripts) $Q_{xx}$ can be written as

(11) $$Q_{xx}=\left[\begin{array}{ccc} Q_{pp} & Q_{pc} & Q_{pa}\\ Q_{cp} & Q_{cc} & Q_{ca}\\ Q_{ap} & Q_{ac} & Q_{aa} \end{array}\right]$$

Since we are focusing only on the geometry of the problem, we build the analysis only in the construction of the design matrix A and the inverse of the normal matrix $Q_{xx}$.

### 3.3. PDOP

The effect of the geometry between the receiver and the satellites on the precision of the receiver position estimate can be evaluated using the positioning dilution of precision (PDOP) index. To compute the PDOP, the $Q_{pp}$ is used. In particular, we suppose that the geometry of the problem has been simulated in local topocentric coordinates East, North and Up with respect to the receiver. Therefore, $Q_{pp}$ has the following structure:

(12) $$Q_{\mathrm{pp}}=\left[\begin{array}{ccc} q_{East} & q_{EN} & q_{EU}\\ q_{NE} & q_{North} & q_{NU}\\ q_{UE} & q_{UN} & q_{Up} \end{array}\right]$$

The diagonal elements of this sub-matrix represent the precision indexes of the estimated 3D coordinates. PDOP is defined as:

(13) $$\mathrm{PDOP}=\sqrt{q_{East}+q_{North}+q_{Up}}$$

In a single epoch, PDOP provides a measure of how well distributed the satellites are in the sky. In this simulation of a batch PPP solution, it provides an estimate of the conditioning of the estimate of the coordinates.

## 4. Simulation

The simulated scenario was defined to take place on 15 September 2024, in the city of Milan, Italy, precisely in the permanent GNSS station, located in latitude (ϕ) $45^{\circ }28^{\prime }42.12^{\prime \prime }$ N and longitude (λ) $09^{\circ }13^{\prime }45.16^{\prime \prime }$ E. IGS final products are used for orbit interpolation of the GNSS satellites.

### 4.1. LEO Orbit Determination

The simulated LEO constellation follows a Keplerian orbital model. Thus, the Keplerian orbital parameters were simulated to provide optimal coverage, according to the models already available in the literature [24,32]. LEO satellite orbit is assumed to be circular, meaning eccentricity e=0. As a consequence, the true anomaly ψ(t) is equal to the mean anomaly M(t) at any epoch *t*, and the argument of perigee ω is fixed arbitrarily. For this simulation, the semi-major axis *a* is equal to the radius of the Earth plus the satellite altitude (7570 km), which corresponds an orbital period of *T* = 109 min. We simulated a constellation of 263 satellites, distributed across 19 orbital planes: 8 orbital planes are polar, with an inclination of $89^{\circ }$, and 11 orbital planes have an inclination of $55^{\circ }$. The orbital planes with the same inclination are simulated to be equally spaced. These specific inclination values were selected based on the following considerations:

- A polar inclination provides global coverage, guaranteeing a sufficient number of satellites also in the polar regions.
- A $55^{\circ }$ inclination allows better coverage over mid-latitude regions, which are the most densely inhabited.

Each polar orbit contains 15 satellites, while each $55^{\circ }$ inclined orbit contains 13 satellites. In each one orbital plane, the satellites are equally spaced. Constellation parameters are summarized in Table 1.

### 4.2. Sky Visibility

Assessing sky visibility is necessary for validating the performance of a constellation. Prior to the PPP analysis, we investigate the satellites visibility under different environmental conditions. We analyse the impact of these conditions on the number of visible LEO satellites and compare their availability to that of GNSS satellites.

The simulation included three test cases, corresponding to three different levels of obstructions. This was achieved by imposing different cutoffs on satellites visibility. In particular, the developed scenarios were the following:

1. Open-sky, with a low cutoff angle of 5^∘^.
2. Residential area, with moderate obstructions corresponding to a cutoff angle of 30^∘^.
3. Urban canyons, with a dense urban environment which increases the cutoff angle to 50^∘^.

**Figure 1:**
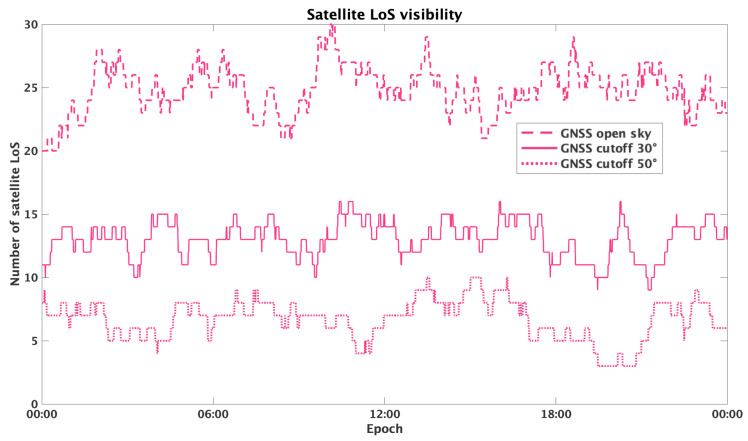
GNSS satellites visibility over the day, for elevation cutoffs of 5^∘^ (dashed line), 30^∘^ (continuous line) and 50^∘^ (dotted line).

**Figure 2:**
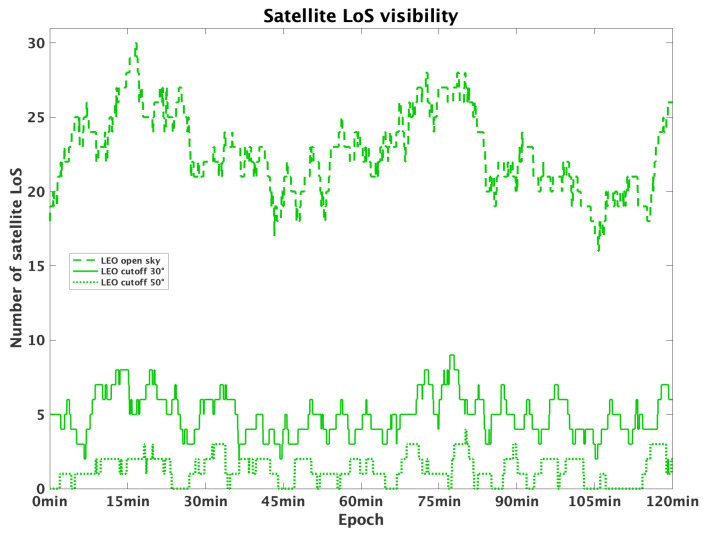
LEO satellites visibility over a 120 min observation period, for elevation cutoffs of 5^∘^ (dashed line), 30^∘^ (continuous line) and 50^∘^ (dotted line).

Sky visibility graphs (Figure 1 and Figure 2) were used to analyse the satellites in line of sight (LoS) over time, for both GNSS and LEO constellations, for the three developed scenarios. GNSS visibility is reported for the whole day. LEO visibility is reported and zoomed just over 2 h, because the constellation visibility repeats itself at about the same period. Table 2 and Table 3 report the statistical analysis of the satellite visibility for both GNSS and LEO constellations. Comparing their performance, we arrive at the following conclusions: GNSS constellation offers a higher visibility in open-sky scenarios with an average of 24 visible satellites; also, the simulated LEO provides a similar number of in view satellites. However, for higher cutoff angles, LEO visibility worsens significantly, while GNSS also offers sufficient visibility in residential and urban conditions.

This analysis underlines the need for integrating multiple satellite constellations to optimize positioning performance, particularly in dense urban environments.

### 4.3. PDOP Analysis Tests in PPP Simulation

The aim of this work is to compare PDOP values obtained by a PPP processing the tested configurations. For the analysis, we have chosen ten static sessions equally distributed over the day, nearly every 2.5 h. The study analysis comprises two primary tests:

1. Test 1: PDOP computation for different sessions lengths. Static sessions are simulated, with duration decreasing from 10 to 5 to 2 min. An improvement, expressed in percentage, is calculated to show the relative decrease in PDOP values when using the hybrid GNSS+LEO constellation compared to GNSS alone. This improvement is calculated as
  (14) $$\mathrm{Improvement}\;(\%)=\left(\frac{{\mathrm{PDOP}}_{\mathrm{GNSS}}-{\mathrm{PDOP}}_{\mathrm{GNSS}+\mathrm{LEO}}}{{\mathrm{PDOP}}_{\mathrm{GNSS}}}\right)\times 100$$
2. Test 2: PDOP convergence analysis in time. This test examines the time needed for a static session to achieve a PDOP value of less than, or equal to, 1.

All the tests are implemented for the three different cutoff angles corresponding to different scenarios.

## 5. Results

### 5.1. Test 1: PDOP Computation for Different Sessions

The analysis of the PDOP values confirms that the integration of LEO satellites improves the overall geometry of the satellites constellation and the quality of the position estimate. The hybrid GNSS+LEO constellation performs better than GNSS-only constellation for all specified sessions (Table 4, Table 5 and Table 6) scenarios.

Observing the results presented in Table 4, Table 5 and Table 6, it is evident the increase in PDOP values as the acquisition period shortens is due to the reduced number of available observables, which in turn prolongs the convergence time of the solution. For the 10 min sessions (Table 7), the integration of LEO significantly improves PDOP by approximately 99.3% for a 5^∘^ cutoff, 93.4% for a 30^∘^ cutoff and 94.4% for a 50^∘^ cutoff. In the 5 min sessions (Table 7), improvements are similarly high, reaching 99.6% at a $5^{\circ }$ cutoff, 98.9% at a $30^{\circ }$ cutoff and 82.1% at a 50^∘^ cutoff. Finally, for the 2 min sessions (Table 7), the GNSS+LEO constellation maintains a good improvement for lower cutoff angles, achieving 99.6% for a 5^∘^ cutoff and 99.5% for a 30^∘^ cutoff. However, at a 50^∘^ cutoff, the improvement is not significant. This was expected, because the number of LEO satellites is very low.

These results confirm that the GNSS+LEO hybrid constellation can significantly enhance satellite geometry, especially in open-sky conditions or with small cutoff angles. To obtain improvements in urban canyon scenarios, a more optimized LEO constellation needs to be studied.

### 5.2. Test 2: PDOP Convergence Analysis in Time

**Figure 3:**
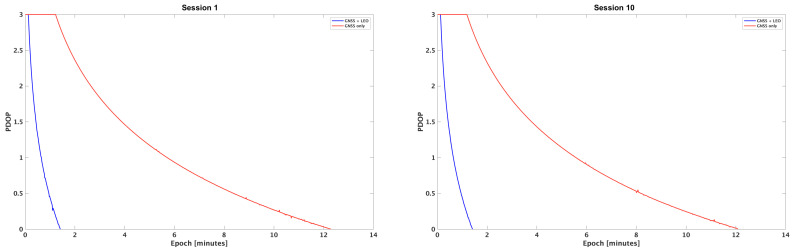
PDOP trend over time (logarithmic scale) for the first and last sessions with a cutoff angle of 5^∘^, comparing GNSS-only (red) and GNSS+LEO (blue) configurations. Values greater than 1000 are capped at 1000 to improve readability.

Firstly, we analyse the PDOP behavior in time for the ten sessions. As expected, in all of them it decreases in time, more slowly for GNSS alone, more quickly for the hybrid configuration. To fix a value for defining a fair solution, i.e., the convergence time, we define a PDOP threshold as (PDOP≤1). As an example, Figure 3 shows the PDOP trend over time on a logarithmic scale for a cutoff angle of 5^∘^ during the first and last sessions analyzed. Please note that, in the plots, initial PDOP values greater than 1000 are not plotted and set to 1000 to improve readability; plots stop when the PDOP reach 1. This conclusion reinforces the results from Test 1: GNSS+LEO configurations can more quickly reach high-precision positioning, especially in favorable conditions. For the other sessions, the results are completely similar and are summarized for all the cutoff angles in Table 8.

The addition of LEO satellites demonstrates a consistent reduction in convergence time (PDOP≤1) across all cutoff angles. For instance, in scenarios with a 5^∘^ cutoff, the mean improvement reaches 83.8%, while for higher cutoff angles, the improvement percentages are somewhat lower, at 83.4% for 30^∘^ and 66.1% for 50^∘^ cutoff angles (Table 9). This confirms that the GNSS+LEO configuration reduces time to achieve acceptable PDOP values, which is particularly beneficial in applications requiring rapid and reliable positioning accuracy.

## 6. Conclusions

This study investigated the possible improvements obtainable in precise point positioning convergence time, provided by the integration of LEO satellites in addition to the existing GNSS constellations. The analysis was carried out for a representative day in 2024 at a test location in Milan, Italy. Two scenarios were considered: one relying only on current available GNSS satellites, and a second that included a simulated LEO-PNT constellation. The LEO constellation used in this study consists of 263 satellites distributed across 19 orbital planes, with an orbital radius of 7570 km. The configuration includes 8 planes inclined at 88^∘^ and 11 planes inclined at 55^∘^, providing a geometry aligned with recent proposals in LEO-PNT research. For the two scenarios, the precision of the PPP solution, quantified using the PDOP index, is evaluated for varying session lengths throughout the day.To represent different obstruction configurations, simulations were performed under three elevation cutoff angle scenarios: open-sky (5^∘^), residential (30^∘^) and urban canyons (50^∘^). In open-sky and residential scenarios, the introduction of LEO satellites led to a substantial improvement in short-session geometry. The time required to reach a satisfactory PDOP value was consistently reduced to under 3 min, compared to 10–15 min for the GNSS-only configuration. For the given simulation, in urban canyons the number of LEO satellites is generally small, but in any case improvements are obtained with respect to a GNSS-only configuration. It is expected that better results could be achieved with more optimized LEO constellation designs. Future work will be focused on the simulation of LEO observations and the optimization of the LEO constellation, with a specific concern for PNT applications for dense urban scenarios in Europe.

## Figures and Tables

**Table 1:** LEO constellation design parameters.

Inclination	Nr. Orbital Planes	Nr. Satellites per Orbital Plane	Period
89^∘^	8	15	109 min
55^∘^	11	13	109 min

**Table 2:** Min, max and mean number of visible GNSS satellites, at different elevation cutoffs: 5^∘^, 30^∘^, and 50^∘^.

GNSS	Cutoff 5^∘^	Cutoff 30^∘^	Cutoff 50^∘^
Min	20	9	3
Max	30	16	10
Mean	25	11	6

**Table 3:** Min, max and mean number of visible LEO satellites, at different elevation cutoffs: 5^∘^, 30^∘^, and 50^∘^.

LEO-PNT	Cutoff 5^∘^	Cutoff 30^∘^	Cutoff 50^∘^
Min	16	2	0
Max	30	9	4
Mean	23	5	1

**Table 4:** PDOP values for GNSS and GNSS+LEO constellations; sessions of 10 min for the three cutoff scenarios.

Session	Cutoff 5^∘^			Cutoff 30^∘^			Cutoff 50^∘^	
	GNSS	GNSS+LEO		GNSS	GNSS+LEO		GNSS	GNSS+LEO
1	2.04	0.01		2.81	0.06		4.59	2.44
2	1.47	0.01		2.64	0.06		49.76	1.18
3	1.49	0.01		3.06	0.07		43.01	1.18
4	1.86	0.01		2.81	0.06		10.51	1.10
5	1.46	0.01		3.02	0.04		7.25	0.98
6	1.44	0.01		2.22	0.06		21.05	1.22
7	1.37	0.01		2.37	0.06		23.11	1.10
8	1.69	0.01		3.19	1.37		6.98	2.21
9	1.69	0.05		3.66	0.05		37.50	1.25
10	1.63	0.01		2.89	0.06		39.82	1.05
**Mean**	1.61	0.01		2.87	0.19		24.36	1.37

**Table 5:** PDOP values for GNSS and GNSS+LEO constellations; sessions of 5 min for the three cutoff scenarios.

Session	Cutoff 5^∘^			Cutoff 30^∘^			Cutoff 50^∘^	
	GNSS	GNSS+LEO		GNSS	GNSS+LEO		GNSS	GNSS+LEO
1	16.17	0.05		22.30	0.29		44.85	24.41
2	11.76	0.05		21.75	0.27		407.49	35.93
3	11.43	0.05		23.76	0.25		193.24	34.64
4	14.60	0.05		22.39	0.25		71.98	42.90
5	11.63	0.05		22.83	0.26		50.14	36.19
6	11.22	0.05		16.29	0.23		181.10	35.62
7	10.88	0.05		17.85	0.30		244.87	14.32
8	13.35	0.05		26.16	0.27		60.02	30.39
9	13.32	0.05		28.64	0.14		338.33	65.75
10	12.84	0.05		19.82	0.24		423.48	39.63
**Mean**	12.72	0.05		22.18	0.25		201.55	35.98

**Table 6:** PDOP values for GNSS and GNSS+LEO constellations; sessions of 2 min for the three cutoff scenarios.

Session	Cutoff 5^∘^			Cutoff 30^∘^			Cutoff 50^∘^	
	GNSS	GNSS+LEO		GNSS	GNSS+LEO		GNSS	GNSS+LEO
1	250.0	0.74		344.82	1.71		676.8	625.4
2	182.9	0.74		344.74	2.05		6311.9	6023.2
3	175.4	0.69		368.27	1.79		2927.0	2728.7
4	228.6	0.75		191.13	1.72		1083.1	1033.1
5	179.2	0.74		355.24	1.81		775.9	765.6
6	173.0	0.73		252.22	1.78		2757.2	2745.7
7	167.1	0.78		298.16	1.74		4987.9	4939.2
8	206.6	0.78		404.65	1.83		947.8	941.7
9	205.8	0.74		440.35	1.54		5604.1	5604.7
10	187.8	0.74		313.48	1.81		10,900.2	10,868.9
**Mean**	195.6	0.70		331.3	1.8		3697.2	3627.6

**Table 7:** Percentage improvement in PDOP values when integrating LEO satellites with GNSS, for different session durations and cutoff angles.

Cutoff Angle	Session Duration		
	10 min	5 min	2 min
5^∘^	99.3%	99.6%	99.6%
30^∘^	93.4%	98.9%	99.5%
50^∘^	94.4%	82.1%	1.8%

**Table 8:** Minimum time (minutes) required to reach PDOP≤1 for GNSS and GNSS+LEO constellations, for the three specified scenarios.

Session	Cutoff 5^∘^			Cutoff 30^∘^			Cutoff 50^∘^	
	GNSS	GNSS+LEO		GNSS	GNSS+LEO		GNSS	GNSS+LEO
1	12.6	1.8		11.7	2.4		10.0	6.4
2	11.3	1.8		11.9	2.3		11.9	7.2
3	11.2	1.7		12.4	2.2		29.3	9.0
4	11.0	1.8		14.4	2.4		22.4	8.7
5	8.5	1.8		14.0	2.1		32.1	8.2
6	11.1	1.8		13.2	2.3		14.5	9.3
7	11.0	1.8		16.5	2.6		33.4	9.1
8	11.2	1.8		14.6	1.5		35.1	8.4
9	11.5	1.8		13.4	2.6		31.3	7.1
10	12.1	1.8		13.2	1.9		19.3	7.6
**Mean**	11.1	1.8		13.5	2.2		23.9	8.1

**Table 9:** Percentage reduction in the time required to reach PDOP≤1 for GNSS+LEO configuration with respect to GNSS only, for different cutoff angles.

Cutoff Angle	Improvement (%)
5^∘^	83.8%
30^∘^	83.4%
50^∘^	66.1%

## Data Availability

The only data used are IGS GNSS ephemerides, available at https://igs.org/ (accessed on 10 July 2025).

## Abbreviations

DOP	Dilution of precision
EKF	Extended Kalman filter
GEO	Geostationary Earth orbit
GNSS	Global navigation satellite system
HAS	High-accuracy service
IGS	International GNSS service
IGSO	Inclined GeoSynchronous Orbit
ISB	Inter-system bias
LEO	Low Earth Orbit
LoS	Line of sight
LS	Least squares
MEO	Medium Earth Orbit
PDOP	Positional dilution of precision
PNT	Positioning, navigation and timing
PPP	Precise point positioning
SPP	Standard point positioning
RTK	Real-time kinematic
